# Resveratrol supplementation enhanced SSRIs efficacy in premenopausal women with major depressive disorder

**DOI:** 10.1192/j.eurpsy.2023.519

**Published:** 2023-07-19

**Authors:** J. Fedotova, V. Tseilikman

**Affiliations:** ^1^Neuroendocrinology, I. P. Pavlov Institute of Physiology RASci, St. Petersburg; ^2^South Ural State University, Chelyabinsk, Russian Federation

## Abstract

**Introduction:**

Premenopausal period is characterized by cognitive and mood disorders in women (Weber et al. J. Steroid Biochem. Mol. Biol. 2014;142:90–98). Resveratrol (3,5,4′-trihydroxy-trans-stilbene) is a phytoestrogen present in the skin of a range of foods including red grapes, blueberries and peanuts. Resveratrol can act through multiple mechanisms, including binding and activation of estrogen receptors (ER), to increase nitric oxide bioavailability and thereby facilitate the endothelium-dependent vasodilatation necessary for adequate cerebral perfusion (Xia et al. Molecules. 2014;19:16102–16121). Some evidences indicate that resveratrol can improve cognitive processes and emotional state (Kodali et al. Sci. Rep. 2015;5:8075).

**Objectives:**

The aim of the present study was to compare the efficacy the combined treatmnet of SSRIs (vortexine, escitalopram, sertraline and fluoxetine) plus resveratrol (50 mg twice per day) for 6 months therapy on the affective profile of premenopausal woman with clinically confirmed Major Depressive Disorder (MDD)

**Methods:**

For the assessment of affective profile in premenopausal women (35-45 years) with clinically confirmed MDD, we used the different tests: Montgomery-Asberg Depression Rating Scale (MADRS) and Shihan Anxiety Scale (ShARS Scale).

**Results:**

After 6 months of SSRIs plus resveratrol therapy, MADRS Scale showed more significant improvement of the depressive symptoms in premenopausal women with clinically confirmed MDD compared to the SSRIs treatment alone (p>0,05). Moreover, these patients demonstrated a significantl low anxiety state using ShARS Scale.

**Conclusions:**

Thus, our pilot clinical study clearly demonstrated that co-treatment with SSRIs plus resveratrol (50 mg twice per day) was able to enhance the therapeutic effects of SSRIs on the affective-related symptoms in premenopausal women. We need to create new approaches to treat the premenopausal women with MDD using a combination of SSRIs with resveratrol.

**Disclosure of Interest:**

None Declared

